# Non-Invasive Systems Application in Traumatic Brain Injury Rehabilitation

**DOI:** 10.3390/brainsci13111594

**Published:** 2023-11-15

**Authors:** Livia Livinț Popa, Diana Chira, Ștefan Strilciuc, Dafin F. Mureșanu

**Affiliations:** 1 RoNeuro Institute for Neurological Research and Diagnostic, 400364 Cluj-Napoca, Romania; livia.popa@brainscience.ro (L.L.P.); dafinm@ssnn.ro (D.F.M.); 2 Department of Neuroscience, Iuliu Hatieganu University of Medicine and Pharmacy, 400083 Cluj-Napoca, Romania; 3 Research Center for Functional Genomics, Biomedicine, and Translational Medicine, Iuliu Hațieganu University of Medicine and Pharmacy, 400012 Cluj-Napoca, Romania

**Keywords:** traumatic brain injury, rehabilitation, non-invasive technologies

## Abstract

Traumatic brain injury (TBI) is a significant public health concern, often leading to long-lasting impairments in cognitive, motor and sensory functions. The rapid development of non-invasive systems has revolutionized the field of TBI rehabilitation by offering modern and effective interventions. This narrative review explores the application of non-invasive technologies, including electroencephalography (EEG), quantitative electroencephalography (qEEG), brain–computer interface (BCI), eye tracking, near-infrared spectroscopy (NIRS), functional near-infrared spectroscopy (fNIRS), magnetic resonance imaging (MRI), functional magnetic resonance imaging (fMRI), magnetoencephalography (MEG), and transcranial magnetic stimulation (TMS) in assessing TBI consequences, and repetitive transcranial magnetic stimulation (rTMS), low-level laser therapy (LLLT), neurofeedback, transcranial direct current stimulation (tDCS), transcranial alternative current stimulation (tACS) and virtual reality (VR) as therapeutic approaches for TBI rehabilitation. In pursuit of advancing TBI rehabilitation, this narrative review highlights the promising potential of non-invasive technologies. We emphasize the need for future research and clinical trials to elucidate their mechanisms of action, refine treatment protocols, and ensure their widespread adoption in TBI rehabilitation settings.

## 1. Introduction

Traumatic Brain Injury (TBI) is a complex neurological condition that poses significant challenges for rehabilitation. The recorded incidence rates within the populations outside of the European Union range from 811 to 979 per 100,000 individuals annually. Simultaneously, the European Union displayed hospital discharge rates of 287.2 per 100,000 people per year, showcasing notable diversity among its member states [1]. Before the onset of the COVID-19 pandemic, the Global Burden of Disease (GBD) documented a worldwide age-standardized traumatic brain injury (TBI) incidence of 369 per 100,000 people in 2016. In 2019, the updated rates showed a slight decrease to 346 per 100,000 people [2,3]. TBI is initiated with a direct impact causing immediate damage to the brain, followed by secondary processes like inflammation and oxidative stress. These mechanisms lead to further tissue damage and neurological issues. Disruption of the blood–brain barrier can result in swelling and increased pressure inside the skull, worsening the injury. The release of excitatory neurotransmitters may cause neuronal cell death. These complex processes highlight the severity of TBIs, impacting patient outcomes and treatment approaches [4].

Traditional approaches to TBI rehabilitation have predominantly focused on conventional therapies such as physical, occupational, and speech therapy [5]. However, the emergence of non-invasive technologies has opened up new avenues for assessment and intervention in TBI rehabilitation [6]. This paper explores the application of various non-invasive systems, encompassing electroencephalography (EEG), quantitative electroencephalography (qEEG), brain–computer interface (BCI), eye tracking, near-infrared spectroscopy (NIRS), functional near-infrared spectroscopy (fNIRS), magnetic resonance imaging (MRI), functional magnetic resonance imaging (fMRI), magnetoencephalography (MEG), transcranial magnetic stimulation (TMS), and other innovative techniques.

The first part of this paper focuses on the utilization of non-invasive technologies for assessing the consequences of TBI. These technologies enable researchers and clinicians to gain valuable insights into the neural abnormalities, functional impairments, and cognitive alterations associated with TBI. In the second part of this paper, we delve into the therapeutic applications of non-invasive technologies in TBI rehabilitation. Repetitive transcranial magnetic stimulation, low-level laser therapy, neurofeedback, transcranial direct current stimulation (tDCS), transcranial alternative current stimulation (tACS), and virtual reality (VR) are discussed as promising approaches for promoting recovery and functional restoration in individuals with TBI. These interventions leverage the principles of neuroplasticity and aim to modulate neural activity, enhance cognitive functions, facilitate motor recovery, and alleviate symptoms associated with TBI.

By exploring the potential of these non-invasive systems in both assessment and intervention, this paper highlights the impact that technological advancements can have on TBI rehabilitation.

## 2. Non-Invasive Methods for TBI Assessment

Traumatic brain injury (TBI) implies injuries to the neuronal network and recovery from it involves the forming of new circuits between surviving and damaged neurons, generating additional functional states and new gene expression profiles. Monitoring this process can provide important data on the clinical state and on the extent of damage in individuals affected by this “silent epidemic” [7].

Nowadays, there are various non-invasive methods being investigated and put into practice to gather essential data regarding the physical and functional impact of cerebral injury that can lead to accurate diagnoses, triaging and treatment plans.

### 2.1. Electroencephalography (EEG)

In the context of the various electrophysiological changes triggered by TBI, electroencephalography (EEG) is a fundamental assessment method, as it mirrors the synaptic neuronal activity within a designated area of the brain, where electrodes are placed for recording and signaling eventual alteration to the various systems regulating cortical activity.

EEG has been shown to have multiple functions in evaluating TBI patients, like tracking acute and subacute variations in the functioning of the brain, which could be linked to the general clinical state of the individual being monitored and could also contribute to distinguishing between mild and severe TBI and prognostic rates. Acute changes have been detected immediately after mild traumatic brain injury (mTBI), within the first hours, and translated in epileptiform activity on EEG, with diffuse suppression of cortical activity ranging between one and two minutes, resulting in the slowing of the EEG and restoration of the regular baseline in up to an hour afterwards [7]. Studies have revealed that most of these acute EEG anomalies tend to subside in a one-year span. It takes weeks to months for subacute changes to be registered in the form of a higher frequency of the posterior alpha rhythm, considered to be a restoration of the original baseline, after the slowing caused by the trauma [8]. It is also important to mention that EEG is especially valuable in cases in which patients are not responsive, so brain functions cannot be evaluated by means of observing their behavior [9].

Another essential function of the EEG in evaluating TBI cases is related to the probability of developing chronic epilepsy following moderate or severe trauma. EEG has shown potential in delivering a biomarker that helps identify individuals who are exposed to impairment caused by TBI, being a very helpful instrument in diagnosing and treating post-traumatic epilepsy, a condition with significant impact among brain injury patients. EEG is relevant even in the period following recovery from TBI, being a useful instrument in tracking eventual underlying long-term conditions deriving from it and alterations in cognitive or behavioral patterns that cannot be perceived by the patients or their observers but can be registered by EEG [9].

### 2.2. Quantitative EEG (qEEG)

Quantitative EEG (qEEG) is the software-based alternative to the ideal non-stop visualization and interpretation of EEG measurements of original signals by a professional that cannot be accomplished in daily practice. This monitoring method is used for interpreting EEG recordings and the quantitative trends within them through various techniques, among which we mention spectral analysis, focused on determining the frequency configuration in EEG during an established time frame and coherence measurements, which are used to assess the synchronicity between different cerebral areas in terms of EEG frequency, thus quantifying the consistency of neuronal activity. These two techniques are especially relevant in the context of TBI [10].

Acute qEEG changes were detected in mTBI cases, with an instant decline in the alpha rhythm and a spike in theta and delta waves or in the alpha–theta ratio within the first few hours or the first few weeks, while subacute changes were detected in the following months, up to half a year, involving persistence in increased delta frequency and reduced alpha frequency. Chronic qEEG changes were observed to persist for as long as six months, indicating an increase in the delta frequency band, with reduced alpha wave frequency [10]. But, interestingly, there is also diagnosis potential in qEEG that is not so strictly time-related, as there is data showing the identification of criteria based on which mild and severe TBI can be distinguished by analyzing EEGs from a larger time frame; between fifteen days and four years post-injury [11].

### 2.3. Magnetoencephalography (MEG)

Magnetoencephalography (MEG) is another efficient method for evaluating TBI parameters, as it is a non-invasive and accurate instrument for supporting diagnosis [12]. It offers an alternative compared to both scalp and implanted EEG recordings. The essential element of brain function involves the flow of electrical currents within groups of neurons. The MEG sensor array accurately captures the magnetic fields generated by these electric currents within the brain. In contrast to electric fields, magnetic fields do not exert any influence on cerebral parenchyma [13]. Consequently, it is easier to localize and measure the currents responsible for the observed magnetic field. In nearly all MEG investigations, it is assumed that the neuroelectric sources behind the recorded magnetic fields result from the collective postsynaptic currents within the cerebral cortex. Due to this, the majority of source-level analyses focus on identifying neuroelectric currents within the cortex, with occasional reports noting the localization of neuroelectric dipoles in the white matter [14]. The analysis is performed and the results are interpreted on a regional basis, tracking neuroelectric tonus in subcortical, cortical and deep white matter areas of the brain. Tonic variation in network functioning registered by MEG measurements and validated by re-testing is of great utility in diagnosing and evaluating treatment efficacy in TBI, as shown by recent studies [12,15].

Measures taken on tonic neuroelectric activation through MEG were proven to be effective in identifying specific symptoms and clinical syndromes related to TBI. MEG-based analysis of neuronal activity on certain brain regions revealed sensitivity to the existence of insomnia, depression, anxiety, somatization, psychological health, chronic pain, sleep, vestibular, oculomotor and cognitive dysfunction, demonstrating great potential in becoming a valid biomarker for TBI [15].

Results arising from MEG measurements indicate that a distinction can be made between individuals with and without symptoms and there is an accurate recurrence in the measures made on an individual; also, the neuroelectric activity recorded within a cortical region can be distinguished from the rim of white matter. All these findings, considered together, lead to the conclusion that MEG measures could to be reliable biomarkers for TBI and useful instruments for diagnosing and evaluating the treatment of common sequelae deriving from it.

### 2.4. Brain-Computer Interface (BCI)

There is evidence pointing out a strong link between dramatic changes in functional connectivity and TBI, with recent studies showing changes in the correlations between neuronal networks during all the phases (subacute, acute, or chronic) and all the levels (mild, moderate, or severe) of the injury. TBI has been classified as a hyperconnectivity syndrome, because the reaction that is triggered to the lower efficiency of the neuronal networks is an increased level of connectivity [16] and this is the reason why the brain–computer interface (BCI) can be an important tool in assessing the level of damage, supporting diagnosis and therapy.

Diffuse axonal injury (DAI) is known to be a very clear indicator of TBI, with a heavy and direct impact on white matter tracts and, consequently, on brain connectivity. DAI is a marker for TBI because it is a direct result of the accelerating and decelerating forces impacting the brain when trauma takes place, so it cannot be considered a secondary effect that can be associated with inflammation, ischemia or any other sort of brain damage. The cerebral areas most commonly impacted by DAI have been observed to be the corpus callosum, cerebellum and fornix, together with subcortical long-range white matter tracts, while focal lesions typically affect frontal, temporal, occipital and parietal lobules [17]. The presence and the extent of alteration in short-range and long-range white matter connections can be evaluated through the recording of the neuronal connectivity within certain cerebral areas, using regular BCI approaches, thus leading to the BCI’s potential of becoming a very useful tool in diagnosing TBI and its underlying impairments. Devices that can assess and modulate brain connectivity include external closed-loop systems, such as fMRI-neurofeedback and EEG-based neurofeedback therapy (EEG-NFT), as well as internal closed-loop systems like Deep Brain Stimulation (DBS), promoting plasticity in cortico-cortical and cortico-subcortical connections [16].

BCIs are evolving as potential means to replace the brain’s conventional output pathways through sensory organs, peripheral nerves and muscles. This advancement opens up new avenues for communication and computer control in individuals with sensory or motor deficits [18,19]. BCIs have the capability to translate brain signals, acquired through both non-invasive and invasive techniques, into control signals for external devices like computer cursors or robotic limbs [20]. Numerous clinical studies have shown the effectiveness of BCIs in TBI rehabilitation [21,22]. One innovative approach is to apply Hopfield neuronal networks (via cerebral organoids and external microelectronics) in order to prevent memory loss in TBI patients [19,23]. Moreover, the integration of BCIs with functional electrical stimulation (FES) technologies has shown promise in enhancing treatment outcomes [24]. Despite notable advancements in BCI technology, it continues to grapple with several challenges. These challenges encompass concerns such as signal degradation (stemming from implanted recording electrodes), the enduring accuracy and reliability of neural decoding algorithms, the downsizing of the system, potential adverse events linked to FES, and the overall user-friendliness of the entire system, among other factors. Consequently, further exploration is imperative for the application of BCI in TBI rehabilitation [19].

### 2.5. Eye Tracking

The movements of the eye are a great indicator of the brain gathering and processing information, so tracking these movements is thought to be of great utility in assessing TBI subjects. Nowadays, technology allows specialists to perform eye tracking evaluations through non-invasive methods, using small-scale infrared camera systems [25], enabling them to examine fast eye movements (saccades), slower movements to follow a moving stimulus (smooth pursuits), resetting movements which are repetitive and uncontrolled (nystagmus) and pauses on designated areas of interest (fixations). Defining the pattern of these movements, while considering some variations limits for classifying the spatial-temporal and kinematic outcome, like timing, duration, velocity, acceleration, latency and frequency, provides considerable insight on the neurological state of patients, with great potential in revealing deficits in visual, motor or cognitive functions, as well as impairment [26].

Eye tracking could increase the chances of fast detection of TBI where neuroimaging and clinical assessments are limited, given the variety of brain regions and neuronal circuits involved in eye movement control and vision processing, thus having a higher probability of intercepting eventual disruptions and issues.

Even though there is still much space for improvement methodology-wise, the existing literature offers evidence of impairment in all aspects of eye movements after mTBI, showing that saccades, fixations, smooth pursuits and nystagmus are affected by injury. Establishing some reliable methods and instruments could render eye tracking really quite effective in evaluating TBI [26].

Furthermore, scientists are taking the next step and looking into the connection between eye movements and memory, studying the “context effect” (CE), in which several cognitive processes are involved: memory of a target (an item to be recognized), association of context (recalling background information related to the target) and ensemble (merging of the target with the context). Eye tracking is useful in memory testing through the information provided by fixations, as there is evidence that the number of fixations in the learning step can anticipate the probability of recognition in the test step [27]. Fixations can be relevant indicators for the attention level and the dwelling time on target (DTOT) in the encoding phase, as they prove themselves to be pivotal in assessing subjects, indicating that selective attention is impaired following traumatic injury, outlining issues in focusing on the target information and highlighting that the overall memory for faces is damaged post-TBI.

### 2.6. Near-Infrared Spectroscopy (NIRS)

Another method from the optical field thought to be of great potential in evaluating TBI is near-infrared spectroscopy (NIRS), a non-invasive method that measures brain oxygenation fluctuations. Through the use of wavelengths between 700 and 1000 nm, called near-infrared (NIR), and capable of penetrating the skull and cerebral tissue, this method measures the light absorption ratio, as this is known to be directly proportional to the concentration of the chromophore. The degree of light absorption offers valuable indications about regional cerebral oxygenation saturation (rSO_2_) and about the extent to which the delivered oxygen is actually used [28].

Practically, rSO_2_ deriving from NIRS corresponds to jugular bulb venous saturation (SjO_2_) [29]. Since the internal jugular veins are the pathway through which deoxygenated blood and non-absorbed oxygen return to the heart, measuring the level of SjO_2_ provides insight into cerebral perfusion, based on the present or missing equilibrium between cerebral blood flow (CBF) and the metabolic requirement (CMRO_2_), on which constant arterial oxygenation relies. Research has shown that following TBI, this balance is lost and evaluating the SjO_2_ level through NIRS can indicate the degree of damage (e.g., SjO_2_ below 50% indicates that the oxygen supply cannot meet the much higher metabolic demand, so at least 13% of the brain has already become ischemic) [28].

NIRS is also relevant in assessing the brain’s performance in autoregulation after TBI, evaluating if and to what degree variations in cerebral perfusion pressure, blood pressure, blood viscosity, hematocrit or partial pressure of arterial oxygen disrupt the continuous and independent CBF and appropriate oxygen supply, which should be maintained as stable by cerebral circulation under normal circumstances through cerebral vascular reactivity (CVR), which can induce either vasodilatation or vasoconstriction. After TBI, blood pressure is prone to drop because of eventual haemorrhages and blood loss through extracranial injuries, so the body attempts to compensate by keeping a continuous CBF and sufficient oxygen delivered by triggering systemic vasoconstriction. NIRS has been presented by research to be of great promise in evaluating autoregulation capacity, measuring cerebral hemodynamics, like oxygen saturation and CVR, thus predicting the level of trauma (e.g., CBF lower than 20 mL/100 g/min indicates that the brain becomes ischemic) [28].

### 2.7. Functional Near-Infrared Spectroscopy (fNIRS)

Functional near-infrared spectroscopy (fNIRS) is a very useful method for assessing motor function after brain injury, enabling specialists to track relevant biomarkers for evaluating the hemodynamic reaction in the motor cortex and being very efficient in distinguishing real movements from imaginary ones. fNIRS method’s root is found in the notable hemispheric lateralization shown by the spatial dynamics that are activated during blood oxygenation and deoxygenation during motor performance involving the hands, for instance [30]. There are certain characteristics of time-spatial activity within the neuronal links of the brain cortex and certain features of the synergy between the various brain areas that can be monitored in order to obtain significant insight into the integrity of the cognitive function and into the general state of the nervous system.

The great benefit of using fNIRS for assessing TBI patients is that it can assess a variety of cognitive processes, like attention, thinking, memory and social cognition, but especially its potential to target the performance of the executive function, an essential indicator of the degree in which these patients face cognitive impairment, because research shows that this would imply a greater risk for Alzheimer’s disease development [31]. This non-invasive monitoring method consists of placing an optical array composed of infrared light transmitters and sensors that measure the degree of light absorption, covering certain areas of the brain, with the purpose of recording the neuronal activation while the subjects perform given tasks. This assessment implies measuring changes in CBF and the degree of oxygenation within the hemoglobin (HbO), the carrier of both oxygen from the lungs to the brain tissue and the returning carbon dioxide.

There are already several studies highlighting the utility and efficiency of fNIRS in TBI deterioration assessment, showing that a reduced activation strength and a higher activation range in brain activation maps can be observed in subjects post-TBI, revealing the probability of executive dysfunctions. This is why it could be regarded as a highly promising tool for assessing the different types of neurocognitive disorders and impairment degrees caused by traumatic injury.

### 2.8. Magnetic Resonance Imaging (MRI)

Current research also draws attention to neuroimaging methods as reliable strategies for assessing brain injury. Among them, magnetic resonance imaging (MRI) is especially relevant for its potential in investigating hypoxic injury, small contusions and axonal shear, thanks to its precision in detecting almost all forms of intracranial lesions and in performing volumetric measurements [32]. In the context of almost 29% of mTBI going undetected by computed tomography (CT), MRI can prevent aggravations and undertreatment for the subjects affected by it, as well as delay in recovery or post-traumatic stress disorder and major depressive disorder [33].

This non-invasive imaging method is based on very strong magnetic fields that align the protons in the body and detect the energy that is being released while they are returned to the range of that magnetic field after temporarily being pushed against it by a radiofrequency current. The timing and the energy spent on the realignment with the magnetic field provide specialists with essential information about the chemical properties of the studied tissue. There are various MRI techniques, each of them focusing on different features of the brain functioning, and all of them offer a more detailed overview of the brain injury compared to CT, let alone the fact that there is no radiation involved, so no additional risk of further malignancy [34].

### 2.9. Functional MRI (fMRI)

Functional magnetic resonance imaging (fMRI) targets the evaluation of cognitive function through analysis of the hemodynamic response function (HRF) and this renders it very useful in TBI assessment. Its focus is on the blood oxygen level-dependent (BOLD) signal, which is modulated by the link between neural activity and the changes in oxygen levels it causes within hemoglobin. fMRI has been identified as a suitable tool for monitoring spontaneous cerebral activity and the networks involved, especially the connectivity within the default mode network (DMN), which was shown to be affected by TBI and was linked to cognitive fatigue, one of the most frequent symptoms of traumatic injury. There is evidence pointing out a decreased overall functional connectivity in TBI patients, with serious alteration risk even for the strongly linked hub regions like the precuneus or the posterior cingulate cortex [34].

Functional connectivity has been shown to be impacted in both ways by TBI, as various studies reveal. There is evidence indicating hypoconnectivity caused by TBI, with mentions of a decline in functional connectivity inside the thalamus, caudate nucleus and right hippocampus, as well as a lowered number of connections and decreased connection strength within the DMN. Hyperconnectivity has also been registered following TBI and it has been related to attention anomalies caused by an amplified connectivity in the sensorimotor networks. fMRI entails an increased sensitivity to the neurocognitive impairments resulting from fluctuations in structural and functional network connectivity [35].

At the same time, there is evidence arising for fMRI being a valuable resource in forecasting TBI development, starting from statistics of functional connectivity levels and patterns, making the profiling of patients, diagnosis and personalized recovery protocols possible, with promising ability in estimating whether a more intensive cognitive therapy is required, along with more attentive monitoring, or if palliative care is sufficient for a certain subject.

### 2.10. Transcranial Magnetic Stimulation (TMS)

Transcranial magnetic stimulation (TMS) is also thought to be of great value in assessing TBI, and is used for evaluating several features of brain circuitry by inducing electrical currents within the cerebral layers. Both single-pulse (sp-TMS) and paired-pulse TMS (pp-TMS) have shown notable efficacy in investigating corticospinal tract, spinal cord and peripheral nerve integrity, but also in detecting changes in cortical dynamics.

Single-pulse TMS targets the assessment of the motor system by using stimulation intensities capable of generating a motor-evoked potential (MEP) that can provide a review of the state of motor pathways in the brain, including the motor fibers’ features, like integrity, diameter and myelin sheath thickness, but also the number of synapses [36]. Sp-TMS uses multiple outcome variables, among which the most frequent are the motor threshold (MT) (thought to be an indicator for corticospinal tract state), input/output curve (I/O curve) (shown to be of great utility in evaluating cortical elements) and silent period (SP) (relevant for monitoring suppression in the electromyographic activity after the MEP). All of these variables have important roles in assessing TBI and the neurophysiological effect it has on patients. For instance, significant elevation of MT has been observed in TBI, signalling lowered excitability or damage within the corticospinal tract.

Paired-pulse TMS is a valuable evaluation tool for excitatory and inhibitory cortico-cortical connections, providing information regarding specific intracortical processes. Since TBI is strongly linked to cognitive decline correlated with aging, measuring the neurophysiological parameters of the TBI impact on subjects could have an important potential for patients who are more exposed to neurodegenerative conditions and disability. Also, putting in place a TMS protocol for assessing TBI could prove itself effective in the prognostic process, given its potential for providing valuable information on neuronal plasticity. Also, there are several studies revealing cortical disinhibition and relevant changes in cortical excitability in relation to the severity of the diffuse axonal injury and motor dysfunction in patients affected by TBI [36].

## 3. Therapeutic Approaches for TBI Rehabilitation

While traditional rehabilitation techniques have played a crucial role in the recovery process, advancements in technology and neuroscience have opened up new avenues for enhancing TBI rehabilitation outcomes. This section explores several emerging therapeutic approaches that show promise in augmenting the recovery process for individuals with TBI. These approaches include repetitive transcranial magnetic stimulation (rTMS), transcranial direct current stimulation (tDCS), transcranial alternating current stimulation (tACS), low-level laser therapy (LLLT) neurofeedback, and virtual reality (VR) therapy.

This section will delve into the underlying mechanisms and clinical applications of these therapeutic approaches. By exploring the potential benefits and challenges associated with each modality, we aim to provide a comprehensive overview of their utility in TBI rehabilitation and contribute to the development of evidence-based treatment strategies that optimize recovery outcomes for individuals affected by TBI.

### 3.1. Repetitive Transcranial Magnetic Stimulation (rTMS)

Repetitive transcranial magnetic stimulation (rTMS) is a neuromodulation technique that utilizes rapidly oscillating magnetic fields to stimulate neural activity. This is achieved by passing brief electrical currents through a coil, generating a magnetic field that stimulates cortical neurons located beneath the focal point of the coil [37]. rTMS is a flexible technique, and depending on the location and frequency used, can be used to either inhibit or induce local and remote brain activity [38]. rTMS is commonly administered as a series of repetitive pulses with a consistent stimulus interval. High-frequency stimulation, defined as equal to or greater than 5 Hz, is believed to enhance neuronal excitability. On the other hand, low-frequency stimulation, below 1 Hz, is associated with inhibitory effects [39].

rTMS offers a significant advantage in terms of its safety profile and the overall absence of significant adverse side effects [37]. Previously, the occurrence of seizures was a concern associated with rTMS; however, stringent safety guidelines have been established subsequent to their documentation, resulting in an exceedingly low incidence rate of such events. The implementation of these guidelines has effectively mitigated the risk and rendered seizures an extremely rare outcome in the context of rTMS administration [39].

When addressing post-TBI depression and anxiety, the effectiveness of using rTMS alone as an intervention may be limited compared to a comprehensive treatment approach that combines rTMS with cognitive behavioral therapy and/or pharmacological therapy [40].

### 3.2. Low-Level Laser Therapy (LLLT)

Low-level laser therapy (LLLT) is a form of non-invasive brain stimulation (NIBS) that involves the application of specific infrared wavelengths capable of penetrating deep into the brain. This technique triggers a range of biological responses, including the formation of adenosine triphosphate (ATP), the enhancement of DNA and RNA, the release of nitric oxide (NO), the regulation of reactive oxygen species (ROS) and the modification of intracellular organelle membrane activity [41,42,43,44].

These multifaceted effects of LLLT have garnered attention in various medical and therapeutic applications, as they hold promise for promoting cellular health and tissue repair. Low-level laser therapy appears to offer a range of benefits that can be attributed to a multitude of biological mechanisms. Among these mechanisms, LLLT demonstrates neuroprotective properties by limiting the spread of brain cell death that often follows a brain injury, resulting in smaller lesion sizes. Additionally, it exhibits anti-inflammatory and anti-edema effects, potentially aiding its favorable outcomes. Another intriguing potential mechanism is its proangiogenic effects, which can stimulate the formation of new blood vessels in the brain, supporting tissue healing. Moreover, LLLT holds exciting prospects in stimulating neurogenesis, potentially fostering the generation of new brain cells and encouraging existing ones to establish new synaptic connections, a phenomenon known as synaptogenesis or synaptic plasticity. These mechanisms collectively contribute to the therapeutic potential of LLLT, particularly in the context of neuroprotection and neurological rehabilitation, offering hope for improved outcomes in cases of brain injury and related conditions [44].

### 3.3. Transcranial Direct Current Stimulation (tDCS)

Transcranial direct current stimulation (tDCS) is a non-invasive brain stimulation technique that has been extensively investigated for its safety and efficacy in various disorders, including TBI [45]. This technique includes administering a mild electrical current (typically ranging from 1 to 2 mA) through the placement of two electrodes on the head to modulate cortical activity. By affecting the resting membrane potentials of neurons, tDCS enhances the chances of depolarization, resulting in increased cortical excitability, or hyperpolarization, leading to decreased cortical excitability [46].

Anodal and cathodal transcranial direct current stimulation (tDCS) are frequently utilized techniques to respectively augment and diminish cortical excitability. The selection of specific montage configurations and stimulation parameters allows for the precise targeting of distinct cerebral networks, encompassing those intricately involved in cognitive processes and motor activities. By applying anodal tDCS, cortical excitability can be increased, leading to heightened neuronal depolarization and subsequent functional enhancements. Conversely, cathodal tDCS induces a decrease in cortical excitability through hyperpolarization, resulting in a reduction in neuronal activity within the targeted regions. The differential effects of anodal and cathodal tDCS, coupled with the versatility of tDCS configurations, enable researchers to investigate and modulate specific neural networks associated with cognition and motor functions [47].

### 3.4. Transcranial Alternating Current Stimulation (tACS)

Transcranial alternating-current stimulation (tACS) is a non-invasive brain stimulation technique known for its ability to entrain natural brain oscillations at specific frequencies. It offers advantages such as cost-effectiveness, tolerability, portability, and safety, making it a promising tool for enhancing cognitive performance. While research on the cognitive effects of tACS is still relatively new, several studies have indicated its potential to enhance working memory, particularly in older adults and individuals with cognitive impairments [48].

The sensations experienced beneath the electrodes during transcranial alternating-current stimulation are typically milder compared to those felt during transcranial direct-current stimulation (tDCS). This discrepancy can, in part, be attributed to the reduced intensity of electrochemical effects. One could hypothesize that sensory neurons’ cell membranes might function as low-pass filters, making them less responsive to high-frequency signals [49,50].

tACS can be employed to selectively stimulate particular brain regions and frequencies linked to cognitive functions like memory, attention, and executive abilities. It also holds the promise of adjusting neural activity to facilitate neuroplasticity [51]. Nonetheless, it is crucial to underscore that the utilization of tACS in the context of traumatic brain injury (TBI) remains an evolving field of investigation, and its efficacy and safety for TBI patients are subjects of ongoing research.

### 3.5. Neurofeedback

Neurofeedback is a biofeedback method used to train individuals with neurological and psychiatric conditions in altering their brain activity through operant conditioning. This approach enables individuals to acquire the ability to amplify or suppress specific electrophysiological parameters as part of a learning process. Behavioral responses are then adjusted by providing feedback and positive reinforcement to facilitate these changes [52,53].

In many cases, neurofeedback utilizes the patient’s quantitative electroencephalogram (qEEG). This qEEG analysis yields power spectral density measurements for each EEG channel, as well as assessments of “coherence”, which represents the power density correlations between two channels. This extensive dataset includes power and coherence measurements across 64 frequencies for 19 channels, resulting in thousands of potential targets for biofeedback. The selection of these targets is often aided by referencing a normative database constructed from qEEG data obtained from healthy individuals [54].

The effectiveness of neurofeedback intervention in the context of traumatic brain injury (TBI) is reasonably well-established. Positive outcomes have been documented in cases of mild TBI (mTBI) following a regimen of 20 treatment sessions of neurofeedback intervention in conjunction with cognitive retraining [55]. In the context of spontaneous recovery, neurofeedback has demonstrated its utility in improving attention difficulties among individuals with closed head injuries [56]. Neurofeedback has also shown promise in addressing physical balance, incontinence, and swallowing issues in individuals with traumatic brain injuries. This suggests that the intervention technique holds significant potential for improving various aspects of TBI-related conditions [57].

Additionally, stress plays a significant role in the recovery process following a traumatic brain injury. Neurofeedback can offer individuals a mental advantage by enabling them to enter the desired state in which to effectively manage various stressors. Furthermore, in neurofeedback, the operant conditioning of measurable neuronal variables, such as amplitude variations in different brainwave frequencies, has the potential to directly influence the biochemical and physiological foundations of an individual’s stress response. Consequently, this can result in lasting alterations in how a person responds to stressors [58].

### 3.6. Virtual Reality (VR) Therapy

Virtual reality (VR) is a computer technology that creates computer-generated artificial environments featuring unique sensory attributes, allowing real-time interaction. VR tools offer several advantages, including the potential for active learning in engaging activities, the capacity to adjust the task difficulty level, and the ability to assess an individual’s behavior and performance. Furthermore, VR can be customized to align with an individual’s specific treatment goals, offering repetitive exercises and allowing for a gradual increase in task complexity while reducing the need for constant guidance from a clinical therapist [59].

The utilization of VR in healthcare has seen significant growth in recent years, with ongoing exploration driven by the expanding accessibility and continuous technological advancements in this field [60]. Virtual reality shows significant potential as an effective aid in TBI rehabilitation, particularly for treatments targeting movement and motor skills. VR-based rehabilitation offers a secure environment where individuals can practice skills with minimal personal risk. Moreover, certain VR platforms have the capability to simulate a wide range of environments, making them valuable for rehabilitating motor skills like walking, balance, and reaching movements across various terrains [61,62].

Additionally, it holds promise for enhancing cognitive rehabilitation. The integration of immersive virtual reality interventions in neurorehabilitation has proven to be effective in enhancing particular executive functions and increasing information processing speed among patients with brain injuries during the subacute phase [63]. Furthermore, virtual reality has been harnessed as a valuable tool for training and enhancing attention in individuals who have experienced severe traumatic brain injuries [64].

### 3.7. Non-Invasive Therapeutic Approaches in Different Phases of TBI

Traumatic brain injury (TBI) presents a complex clinical challenge in the sequence of cellular reactions following a brain injury unfolding across four distinct phases: the hyperacute phase (lasting from minutes to hours), the acute phase (extending for hours to several days), the post-acute phase (persisting for several days to weeks) and the chronic phase (lasting for months and beyond) [65]. Multifaceted strategies are required to address its acute, sub-acute, and chronic phases. During the hyperacute and acute phase, the immediate priority is patient stabilization and assessment [66]. While therapeutic interventions, particularly non-invasive ones, may have limited application due to pressing medical concerns, early assessments can identify potential candidates for later interventions. In the post-acute phase, a shift toward neurorehabilitation becomes prominent. rTMS holds a better potential for cognitive and motor function recovery, tDCS offers cognitive improvement, tACS exhibits promise in cognitive enhancement and neuroplasticity, neurofeedback is effective in addressing attention difficulties, VR proves valuable for motor skill and cognitive rehabilitation, while LLLT displays synaptogenesis and synaptic plasticity [44,47,56,63,67]. Table 1 presents a comprehensive display of these information for easy reference. The chronic phase necessitates ongoing rehabilitation, often demanding a personalized, extended-term strategy to sustain the advantages provided by these non-invasive methods. Nevertheless, it is essential to note that accessing long-term rehabilitation services, especially those introduced in this paper as being more innovative, may present difficulties. While the acute and sub-acute phases of rehabilitation receive significant attention, the chronic phase, which extends over months and beyond, can often be marked by a scarcity of comprehensive, ongoing services [68,69]. Recognizing and addressing this issue is pivotal step toward ensuring equitable and effective care for all TBI patients, regardless of the phase of their recovery journey. 

## 4. Conclusions

In conclusion, the exploration of therapeutic approaches for traumatic brain injury rehabilitation has revealed a range of promising interventions that have the potential to enhance recovery outcomes for individuals affected by TBI. Repetitive transcranial magnetic stimulation (rTMS), virtual reality (VR), neurofeedback, low-level laser therapy (LLLT), transcranial direct current stimulation (tDCS), and transcranial alternating current stimulation (tACS) have all demonstrated efficacy in targeting various aspects of TBI-related impairments and in promoting neuroplasticity.

While these therapeutic approaches hold great potential, it is important to acknowledge the need for further research and clinical validation to establish their effectiveness, optimize treatment protocols, and understand their underlying mechanisms. Challenges such as individual variability and treatment standardization need to be addressed to ensure consistent and reliable outcomes across diverse TBI populations.

### Future Directions

Looking ahead, the future of TBI rehabilitation is poised to be greatly influenced by the continued exploration of non-invasive therapeutic systems. The interventions we have discussed, such as repetitive transcranial magnetic stimulation (rTMS), virtual reality (VR), neurofeedback, low-level laser therapy (LLLT), transcranial direct current stimulation (tDCS), and transcranial alternating current stimulation (tACS), are just the tip of the iceberg. To advance this field, future research must aim to fine-tune these approaches, identifying the most effective protocols for different TBI subtypes and varying degrees of injury.

Moreover, addressing the issue of individual variability and standardizing treatment regimens is pivotal to ensuring the reproducibility and scalability of these therapies in a real-world clinical setting. Collaborations between healthcare professionals and researchers can pave the way for the development of more accessible non-invasive systems, which will be crucial for patients’ post-discharge care and long-term rehabilitation.

By continuing to explore these therapeutic approaches, healthcare professionals and researchers can contribute to the development of evidence-based treatment strategies that maximize TBI rehabilitation outcomes. Ultimately, the goal of therapeutic interventions for TBI rehabilitation is to optimize functional recovery, improve quality of life, and enhance the overall well-being of individuals who have experienced this condition. Through ongoing research, we can continue to advance the field of TBI rehabilitation and provide individuals with the best possible chances for recovery, reintegration and a meaningful life beyond their injuries.

## Figures and Tables

**Table 1:** Non-invasive therapeutic approaches’ efficiency in different phases of TBI.

Non-Invasive Technology	Acute Phase	Post Acute Phase	Chronic Phase
Repetitive Transcranial Magnetic Stimulation (rTMS)	Neuromodulation Neruoprotection	Notably better efficiency—cognitive and motor function recovery	Long-term, repeated sessions for sustained benefits
Low-Level Laser Therapy (LLLT)	A range of biological responses Limited brain cell death Anti-inflammatory effects	Proangiogenic effects Synaptogenesis and synaptic plasticity	Ongoing treatment for sustained benefits
Transcranial Direct Current Stimulation (tDCS)	Not a primary intervention at this stage—observational role	Cognitive and motor function recovery	Beneficial for long-term management of symptoms
Transcranial Alternating Current Stimulation (tACS)	Not a primary intervention at this stage—potential tool for observation	Cognitive enhancement and neuroplasticity	Beneficial for long-term management of symptoms
Neurofeedback	Not a primary intervention at this stage	Cognitive enhancement, attention improvement	Long-term stress management Physical balance, incontinence, swallowing
Virtual Reality (VR) Therapy	Not a primary intervention at this stage	Movement and motor enhancement Executive functions and processing speed enhancement	Long-term maintenance of motor skills and continued cognitive rehabilitation

## Data Availability

Not applicable.
